# Development and internal validation of a simple risk stratification score for unplanned emergency department revisits within 24 hours after discharge: A retrospective observational study

**DOI:** 10.1371/journal.pone.0357575

**Published:** 2026-09-02

**Authors:** Hidetaka Onda, Yuma Kuramatsu, Toyoaki Yamamoto, Atsunori Nishimatsu, Mami Kaneko, Daichi Tsutsui, Mizuki Kojima, Shinya Higuchi, Shinya Takeuchi, Kingo Nishiyama, Masato Miyauchi

**Affiliations:** Department of Disaster and Emergency Medicine, Kochi University, Nankoku, Kochi, Japan; Pescara General Hospital, ITALY

## Abstract

**Background:**

Identifying patients at risk of very early emergency department revisits may help support discharge decision-making, but the extent to which routinely available index‑visit data can predict unplanned returns within 24 h remains unclear. Prior studies suggest that revisits are heterogeneous and influenced by factors beyond the initial encounter, underscoring the need to clarify—not overstate—the predictive value of simple, pragmatic tools.

**Methods:**

We conducted a single‑center retrospective study of 2,774 emergency department discharge visits in 2024 in which blood tests were performed. The primary outcome was an unplanned revisit within 24 h to the same emergency department. Two physicians independently adjudicated revisits using administrative and chart data. Candidate predictors included arrival mode, initial vital signs, and routinely available laboratory variables. Continuous variables were screened across prespecified percentile‑based cutoffs, and a simple additive score was constructed from dichotomized items. Internal validation used 2,000 bootstrap resamples with full repetition of item selection and cutoff search. Calibration was assessed using the calibration slope, intercept, and Brier score.

**Results:**

There were 91 unplanned revisits (3.28%). Five dichotomized variables were retained: no ambulance use, C‑reactive protein ≥0.39 mg/dL, lymphocyte percentage ≤13.7%, red cell distribution width ≥13.5%, and mean corpuscular volume ≤87 fL. The odds ratio for revisit per 1‑point increase was 1.55 (95% confidence interval, 1.32–1.82). Apparent discrimination was modest (area under the curve, 0.651; 95% confidence interval, 0.592–0.711), with an optimism‑corrected area under the curve of 0.633. Apparent calibration in the derivation cohort was acceptable (slope 1.000; intercept 0.000; Brier score 0.0313). At a ≥ 4‑point threshold, specificity was 92.77% and sensitivity 20.88%.

**Conclusions:**

A five‑item score based on index‑visit information showed a graded association with 24‑h revisit risk but modest predictive performance. This highlights the limitations of prediction using discharge‑time data alone and emphasizes the need for external validation and prospective evaluation before clinical implementation.

## Introduction

Emergency department (ED) revisits have long been examined as potential indicators of care quality, yet accumulating evidence shows that revisit events are heterogeneous and influenced by multiple clinical and non-clinical factors [1]. Although some studies have focused on severe outcomes such as hospitalization or death after a revisit [2,3], the interpretation of revisit-associated outcomes varies across populations and settings [4,5]. As a result, short-term revisits cannot be viewed as a direct reflection of the quality of the index encounter, and their prediction remains inherently challenging.

Several predictors of short-term revisits have been reported, including older age, higher acuity, longer ED length of stay, and prior ED utilization [6]. The clinical linkage between the index visit and the revisit in older adults has also been explored [7]. Abnormal discharge vital signs and physiologic deterioration during the ED stay are associated with adverse post-discharge outcomes [3,8], and clinicians’ subjective assessments may also contribute to the identification of patients at risk of early return [9]. However, patients with clearly abnormal findings at the initial evaluation are often admitted and therefore removed from the population in which revisit prediction is most relevant. Consequently, the predictive value of initial vital signs alone may be attenuated in a discharge-selected cohort.

Recent work has applied machine learning and deep learning to revisit prediction, including models that incorporate time-series vital-sign data or large administrative datasets [10–12]. These approaches have highlighted both the potential and the limitations of prediction, particularly the difficulty of translating complex models into actionable clinical decision-making [11]. In contrast, simple and interpretable tools based on routinely available information may be more feasible in many EDs, especially those in smaller facilities with limited resources. Laboratory-based risk stratification has also been explored in disease-specific contexts, such as urinary tract infections [13], suggesting that common laboratory indices may reflect an underlying vulnerability or residual clinical uncertainty.

Revisits within 24 h after discharge represent a particularly narrow window that is more likely to remain linked to the index assessment and the discharge decision in comparison with broader intervals such as revisits within 72 h [14,15]. However, even very early revisits are heterogeneous events influenced by patient anxiety, evolving symptoms, comorbidity, and social factors [16,17]. Thus, the ability of routine index-visit data to meaningfully predict such events remains uncertain. Clarifying the limits of prediction in this setting is important, both to avoid overreliance on imperfect tools and to inform realistic expectations for risk stratification in routine emergency care.

Given these considerations, we focused on ED visits resulting in discharge, in which blood tests were performed as part of routine clinical assessment. This population represents encounters in which clinicians had sufficient diagnostic uncertainty to obtain laboratory testing but ultimately judged the patient to be suitable for discharge. Because this group may carry residual risk that is not fully captured by vital signs or clinical impression [18], it is a relevant population in which to examine what routinely available data can—and cannot—predict.

The objective of this study was to develop and internally validate a simple, additive score for stratifying the risk of unplanned ED revisits within 24 h after discharge, using information available at the index visit. Rather than aiming to construct a high-performance predictive model, the goal was to provide a transparent assessment of the predictive value and inherent limitations of routinely collected clinical and laboratory data in a discharge-selected cohort.

## Methods

### Ethical considerations

This single‑center retrospective observational study used existing clinical information from our ED. The study protocol was approved by the Ethics Committee of Kochi Medical School on February 5, 2026 (Approval No. 2025‑147). The study adhered to all relevant ethical guidelines as well as the 1964 Declaration of Helsinki and its later amendments. An opt‑out procedure was implemented in accordance with institutional regulations, all data were de‑identified before analysis, and the requirement for written informed consent was waived because of the retrospective secondary‑use design.

### Study design and participants

We retrospectively analyzed all ED visits to our institution between January 1 and December 31, 2024. The ED is a mixed urban–rural emergency care facility with 24‑h physician coverage and an annual census of approximately 30,000 visits. Triage is performed by trained nurses using a standardized institutional triage protocol.

We included discharge visits in which blood tests were performed during the index encounter and the patient was sent home. Discharge visits without blood testing were excluded because the final score was designed to use laboratory variables that are available at the index visit. Visits resulting in hospital admission, transfer to another hospital, or transport to another facility were also excluded.

The unit of analysis was the visit rather than the patient. Repeated visits by the same individual were identified using a unique patient ID, but each visit was analyzed separately because it represented a distinct clinical encounter with its own assessment and discharge decision. No formal sample size calculation was performed; all consecutive eligible discharge visits during the study period were included.

### Data collection and candidate predictors

Data were extracted from the electronic medical record and laboratory information systems. The data were accessed for research purposes on February 16, 2026, after completion of the study period. The authors did not access the data during the study period itself. Identifiable information was present in the source records at the time of data extraction, but all data were de-identified before analysis. Extracted variables included age, sex, mode of arrival, initial vital signs, and laboratory data obtained at the index ED visit. Mode of arrival was categorized as “ambulance use” or “no ambulance use.”

Initial vital signs included body temperature, heart rate, systolic blood pressure, diastolic blood pressure, respiratory rate, and oxygen saturation. Vital signs were recorded only at the time of initial ED evaluation; our institutional workflow does not mandate reassessment at discharge.

Candidate laboratory variables were collected from tests routinely available within our institutional workflow, which include inflammatory, metabolic, renal, hematologic, erythrocyte, and biochemical parameters. Laboratory measurements were performed using automated analyzers in the hospital’s central laboratory, in accordance with the manufacturers’ specifications. C‑reactive protein was available for 2,481 of the 2,774 visits (89.4%), indicating that it was obtained in routine practice in most included visits, although testing was clinician‑directed rather than protocol‑mandated.

Predictor values were extracted from routine index‑visit records, and no formal blinding procedure was used for predictor assessment.

### Outcome definition

The primary outcome was an unplanned revisit to our ED within 24 h after discharge from the index visit. Candidate revisit events were first identified systematically from institutional visit records according to revisit timing and then reviewed using administrative records, including the reservation processing status, together with the electronic medical record.

Two physicians independently classified each candidate return visit as “planned” or “unplanned.” Planned revisits were defined as return visits explicitly scheduled or instructed at the index visit, whereas unplanned revisits were defined as return visits without such prior scheduling. In the same chart review process, the two physicians also assessed whether the return‑visit complaint was the same as or clinically related to that of the index visit. There were no disagreements between the reviewers. Revisit events were identified only within our institution; revisits to other hospitals or clinics were not captured. No formal blinding procedure was used to adjudicate the outcome.

### Handling of vital signs and laboratory variables

Initial vital signs were analyzed as recorded at the time of the index ED evaluation. Laboratory testing was clinician‑directed rather than uniformly mandated; therefore, individual laboratory items were evaluated among visits in which the corresponding test was performed rather than being interpreted uniformly as missing data. No imputation was performed for predictor‑level analyses.

For calculation of the final additive score, unmeasured final score components were coded as absent (0 points) rather than imputed, enabling the score to be calculated for all included visits.

### Statistical analysis

Continuous variables are presented as medians and interquartile ranges and categorical variables as numbers and percentages. For between‑group comparisons, Fisher’s exact test was used for categorical variables and the Wilcoxon rank‑sum test for continuous variables. Comparisons of vital signs were performed among visits in which the value had been recorded, and laboratory analyses were restricted to visits in which the corresponding test had been performed.

Logistic regression analyses were performed to identify factors associated with unplanned ED revisits within 24 h. Variables that were shown to be significant in univariable analyses and those considered clinically relevant were entered into multivariable models. The multivariable analysis was positioned as a reference analysis to account for overlap among candidate items rather than to establish causal independence. Odds ratios (ORs), 95% confidence intervals (CIs), and p-values were reported.

### Score derivation

For score development, we used a small number of dichotomized items to construct a simple additive score. For continuous candidate variables, candidate cutoffs were screened across prespecified percentile points within the observed distribution, specifically from the 5th to the 95th percentiles in 5‑percentile increments. For each candidate cutoff, a 2 × 2 table was constructed, the OR for the unplanned revisit was calculated, and the cutoff yielding the largest OR was retained.

Because there are no universally established clinically meaningful thresholds for predicting unplanned 24‑h revisits using these variables, the primary thresholding strategy was exploratory and data‑driven. Candidate items were screened from routinely available index‑visit variables, and the final item retention was based on the exploratory cutoff‑screening procedure rather than on descriptive p-values alone. This strategy was intended to identify pragmatic dichotomous predictors suitable for constructing a simple bedside additive score rather than to define biologically meaningful clinical thresholds. Retained items were dichotomized and summed as an additive score, with 1 point assigned to each item.

A weighted model based on the regression coefficients for the same dichotomized items was also examined for comparison. Score performance was evaluated using the receiver operating characteristic curve and the area under the curve (AUC) with 95% CIs. The statistically optimal cutoff was determined using the Youden index, and a high‑risk threshold was examined as the lowest score achieving a specificity of at least 90%. Sensitivity, specificity, positive predictive value, negative predictive value, and the trend test across score levels were also calculated.

### Calibration and internal validation

Calibration was assessed using a logistic regression model with the total score as a continuous predictor. Model‑predicted probabilities were compared with observed revisit rates across score levels, and the apparent calibration intercept, calibration slope, and Brier score were estimated in the derivation cohort.

Because this is a derivation study and both the item-selection and cutoff-determination procedures were data‑driven, internal validation was performed using 2,000 bootstrap resamples. In each bootstrap sample, the full score–derivation procedure was repeated, including candidate‑item screening and cutoff search for continuous variables, and the optimism‑corrected AUC was calculated.

### Sensitivity analysis

As a sensitivity analysis, repeated visits by the same patient were addressed using methods that accounted for within‑patient clustering based on the unique patient ID. Additional sensitivity analyses were performed to evaluate the impact of missing-data handling. The original missing-as-absent approach was compared with complete-case analysis and multiple imputation using chained equations. Model discrimination was assessed for each approach using the area under the receiver operating characteristic curve. All tests were two‑sided, a *P*-value of <0.05 was considered to indicate statistical significance, and analyses were performed using StatFlex Plus ver. 7.0 (View Flex Co., Ltd., Osaka, Japan). This study is reported in accordance with the Transparent Reporting of a multivariable prediction model for Individual Prognosis Or Diagnosis (TRIPOD) guideline.

## Results

### Study cohort

During the study period, a total of 3,737 ED visits resulting in discharge were screened for eligibility. Of these, 963 visits were excluded because no blood tests were performed at the index encounter, leaving 2,774 eligible visits for analysis (S1 Fig). These visits corresponded to 2,427 unique patients, among whom 257 (10.6%) had more than one eligible visit. The maximum number of eligible visits for a single patient was eight. Among the 91 unplanned ED revisits within 24 hours, 38 (41.8%) occurred in patients with multiple eligible visits during the study period, whereas 53 (58.2%) occurred in patients with only a single eligible visit. Overall, 91 unplanned revisits within 24 h were identified, yielding an overall revisit rate of 3.28%. Independent adjudication by two physicians resulted in complete agreement, and all unplanned revisits were judged to involve the same or a clinically related complaint as the index visit. The baseline characteristics of the revisit and non‑revisit groups are summarized in Table 1. Among the 91 unplanned revisits, 18 (19.8%) resulted in hospitalization at the return visit.

**Table 1:** Study cohort and baseline characteristics.

Parameter	Value
Included ED discharge visits, n	2,774
Excluded ED discharge visits without blood testing, n	963
Unique patients, n	2427
Patients with more than one eligible visit, n (%)	257 (10.6)
Unplanned revisits within 24 h, n (%)	91 (3.28)
Sex	
Male (%)	1,289 (46.5)
Female (%)	1485 (53.5)
Age, years	69 (49–80)
Ambulance transport, n (%)	1,793 (64.6)
Visit time category	
Weekday daytime, %	65 (2.3)
Weekday night, %	1,311 (47.3)
Holiday/weekend, %	1,398 (50.4)
Blood pressure, mmHg	
Systolic	142 (124–160)
Diastolic	83 (73–93)
Heart rate,/min	82 (72–96)
Respiratory rate,/min	19 (16–22)
SpO_2_, %	97 (96–100)
Body temperature, °C	36.7 (36.4–37.1)

Weekday daytime, 08:30–17:15; weekday night, 17:15–08:30. Holiday/weekend was defined according to the institutional calendar. ED, emergency department.

### Availability of recorded variables

Initial vital signs were not always completely recorded across the cohort. Body temperature, heart rate, systolic blood pressure, diastolic blood pressure, respiratory rate, and oxygen saturation were available in 57.8%, 46.5%, 56.8%, 56.5%, 51.3%, and 56.7% of visits, respectively. In contrast, the laboratory variables retained in the final score were available in most visits: C‑reactive protein in 89.4%, lymphocyte percentage in 96.5%, red cell distribution width in 99.8%, and mean corpuscular volume in 99.9% of visits (S1 Table).

### Between‑group comparisons

Between‑group comparisons of vital signs and laboratory variables are shown in Table 2. None of the initial vital signs differed significantly between the revisit and non‑revisit groups. In contrast, several laboratory variables showed modest but statistically significant differences. In the revisit group, C‑reactive protein levels and red cell distribution width were higher, while lymphocyte percentage and mean corpuscular volume were lower compared with the non-revisit group. These comparisons were descriptive and were not used as the sole basis for score construction.

**Table 2:** Between-group comparison according to unplanned revisit within 24 h after discharge.

Parameter	Revisit -	Revisit +	*P*-value
Cases, n	2683	91	
Male	1239 (46.2)	50 (56.0)	0.109
Age, years	70 (49.5–80.0)	68 (47.0–77.0)	0.322
No ambulance transport, n (%)	930 (34.7)	51 (56.0)	<0.001
Visit time category			0.688
Weekday daytime, %	64 (2.4)	1 (1.1)	
Weekday night, %	1269 (47.3)	42 (46.2)	
Holiday/weekend, %	1350 (50.3)	48 (52.8)	
Blood pressure, mmHg			
Systolic	142 (124–160)	138 (123–158)	0.736
Diastolic	83 (73–93)	84.5 (75–93.5)	0.540
Heart rate,/min	82 (72–96)	86.5 (75–98.3)	0.183
Respiratory rate,/min	19 (16–22)	20 (17–24.3)	0.066
SpO_2_, %	97 (96–99)	98 (95--99)	0.714
Lymphocyte percentage, %	21.2 (12.6–30.7)	19.0 (10.9–25.4)	0.017
Neutrophil fraction, %	70.4 (59.4–80.3)	73.1 (66.5–83.5)	0.021
RBC, × 10^6^/µL	4.23 (3.85–4.63)	4.21 (3.83–4.59)	0.820
RDW, %	13.1 (12.4–14.1)	13.4 (12.8–14.6)	0.008
MCV, fL	92 (89–95)	90 (87–94)	0.010
Hb, g/dL	12.8 (11.4–14.0)	12.7 (11.1–13.9)	0.448
Cre, mg/dL	0.79 (0.65–1.02)	0.76 (0.64–0.99)	0.456
BUN, mg/dL	15.0 (11.5–20.1)	14.8 (10.9–19.6)	0.627
Na, mmol/L	139 (136–140)	138 (136–140)	0.054
K, mmol/L	4.1 (3.8–4.4)	4.1 (3.8–4.4)	0.971
Cl, mmol/L	103 (100–105)	103 (100–105)	0.896
Alb, g/dL	3.9 (3.6–4.3)	3.7 (3.3–4.4)	0.181
Lac, mmol/L	1.7 (1.3–2.3)	2.1 (1.5–2.6)	0.276
CRP, mg/dL	0.17 (0.05–0.97)	0.48 (0.12–1.42)	<0.001

Alb, albumin; BUN, blood urea nitrogen; Cre, creatinine; CRP, C-reactive protein; Hb, hemoglobin; Lac, lactate; MCV, mean corpuscular volume; RBC, red blood cell count; RDW, red cell distribution width (RDW-CV); SpO_2_, oxygen saturation.

### Candidate predictors and score derivation

Exploratory screening identified five dichotomized variables for inclusion in the final score: no ambulance use, C‑reactive protein ≥0.39 mg/dL, lymphocyte percentage ≤13.7%, red cell distribution width ≥13.5%, and mean corpuscular volume ≤87 fL. The prevalence of each dichotomized item is shown in Table 3. Each retained variable was assigned 1 point, resulting in a simple additive score ranging from 0 to 5. It was possible to calculate the score for all 2,774 visits because unmeasured components were coded as “absent.”

**Table 3:** Candidate predictors retained for score derivation and univariable associations with unplanned revisits within 24 h after discharge.

Parameter	Cutoff	Positivity (%)	OR (95% CI)	*P*-value
No ambulance transport	–	35.36	2.40 (1.58–3.66)	<0.001
CRP, mg/dL	≥0.39	34.10	2.12 (1.39–3.22)	<0.001
Lymphocyte percentage, %	≤13.7	27.22	1.62 (1.05–2.50)	0.031
RDW, %	≥13.5	37.17	1.68 (1.11–2.56)	0.015
MCV, fL	≤87	18.75	1.87 (1.18–2.97)	0.009

CRP, C-reactive protein; MCV, mean corpuscular volume; RDW, red cell distribution width (RDW-CV).

### Association between the score and revisit risk

**Fig 1:**
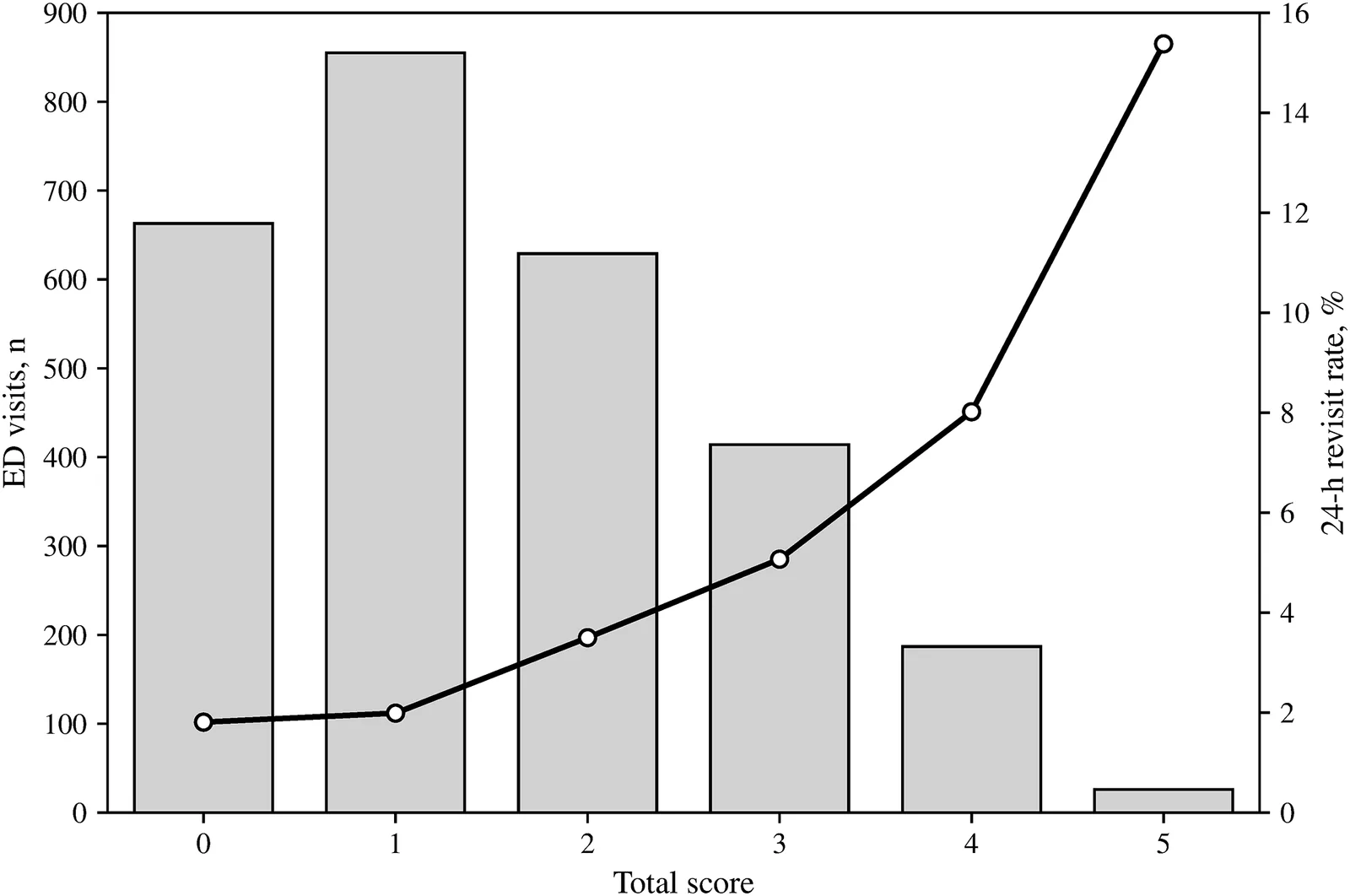
Distribution of total scores and 24-h revisit rate by score level. Bars indicate the number of emergency department visits in each score category, and the solid line indicates the corresponding rate of unplanned revisits within 24 h after discharge. The total score ranged from 0 to 5 points.

Total score demonstrated a graded association with unplanned 24‑h revisits. The OR for revisit per 1‑point increase in score was 1.55 (95% CI, 1.32–1.82). Revisit rates increased across score categories, from 1.81% for a score of 0 to 15.38% for a score of 5. These observed revisit rates are presented in Table 4 and illustrated in Fig 1.

**Table 4:** Revisit rates according to score level and threshold performance.

Panel A. Revisit rates according to score level				
Total score	Revisits/ total	Revisit rate (%)	95% CI	
0	12/ 663	1.81	0.94–3.14	
1	17/ 855	1.99	1.16–3.16	
2	22/ 629	3.50	2.20–5.25	
3	21/ 414	5.07	3.17–7.65	
4	15/ 187	8.02	4.56–12.88	
5	4/ 26	15.38	4.36–34.87	
**Panel B. Threshold performance at selected cutoffs**				
**Cutoff**	**Sensitivity (%)**	**Specificity (%)**	**PPV (%)**	**NPV (%)**
≥2	68.13	55.50	4.94	98.09
≥4	20.88	92.77	8.92	97.19

CI, confidence interval; PPV, positive predictive value; NPV, negative predictive value.

### Threshold performance

The statistically optimal cutoff based on the Youden index was a score of 2 or higher, which yielded a sensitivity of 68.13% and a specificity of 55.50%. A high‑specificity threshold of 4 or higher identified 7.68% of visits and corresponded to a revisit rate of 8.92% within this subgroup compared with 2.81% among visits with scores of 3 or lower. At this threshold, the sensitivity was 20.88% and the specificity was 92.77%. The threshold performance metrics are summarized in Table 4.

### Reference multivariable analysis

When all five dichotomized score components were entered simultaneously into a multivariable logistic regression model, no ambulance use and mean corpuscular volume ≤87 fL remained associated with unplanned revisits after adjustment. The other three laboratory variables did not retain independent associations, suggesting that there was overlap among predictors rather than distinct contributions (S2 Table).

### Sensitivity analysis for repeated visits

A clustered logistic regression model accounting for repeated visits by the same patient produced results similar to the main analysis. The OR for revisit per 1‑point increase in score remained 1.55 (95% CI, 1.32–1.83). In the clustered multivariable model, no ambulance use and mean corpuscular volume ≤87 fL again remained associated with revisit risk. Additional sensitivity analyses evaluating different missing-data handling strategies demonstrated similar discrimination across all approaches. The AUC was 0.651 for the original missing-as-absent analysis, 0.649 for the complete-case analysis, and 0.645 after multiple imputation, indicating minimal influence of missing-data handling on model performance.

### Comparison with a weighted model

A weighted model based on regression coefficients for the same five dichotomized items showed only a minimal improvement in apparent discrimination compared with the unweighted score (AUC 0.663 vs. 0.651), supporting the use of the simpler equal‑weight score.

### Discrimination, calibration, and internal validation

**Fig 2:**
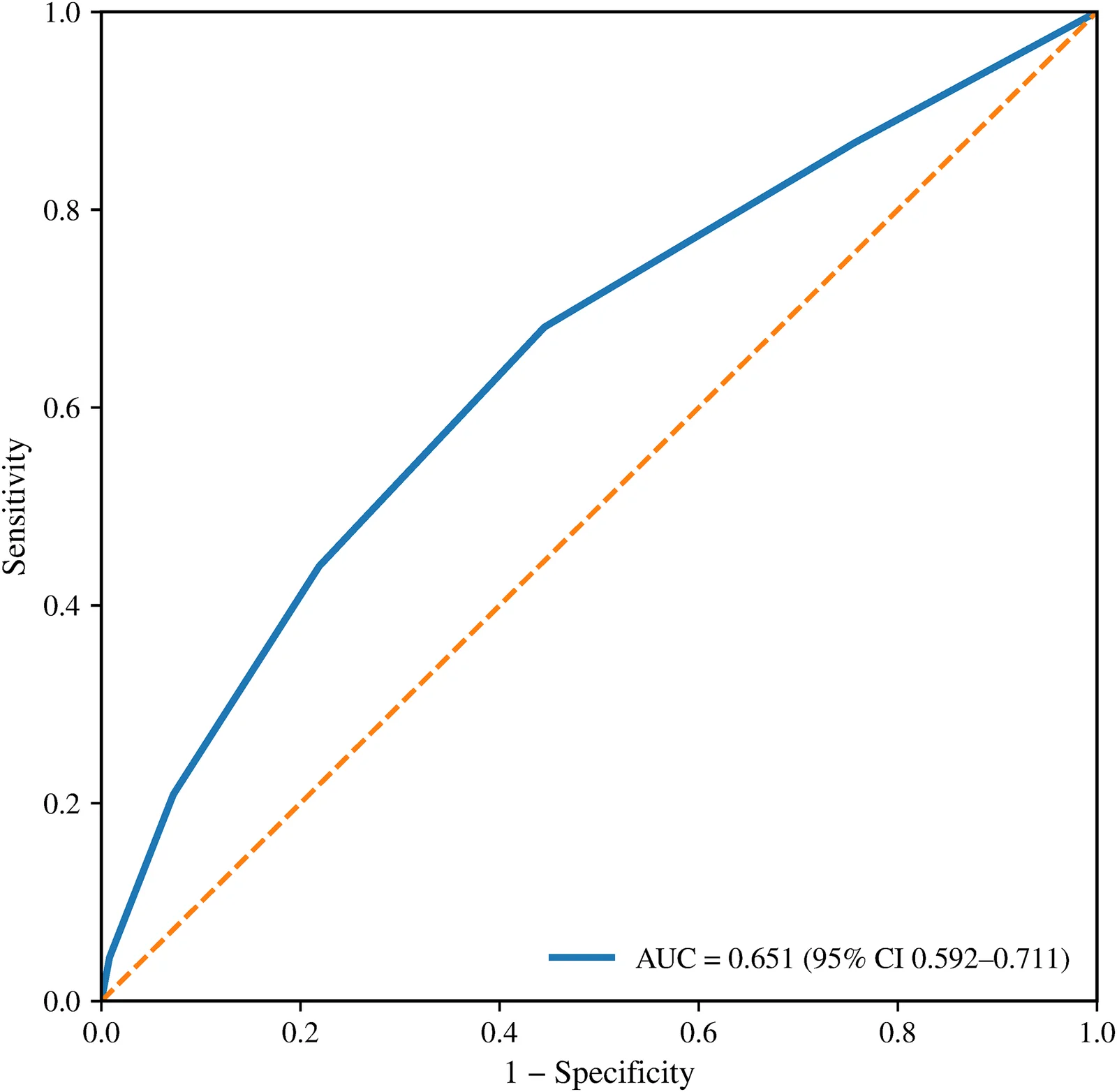
Receiver operating characteristic curve of the five-item score for predicting unplanned 24-h emergency department revisits. The receiver operating characteristic curve shows the discriminative performance of the five-item score for unplanned revisits within 24 h after discharge. The area under the curve (AUC) was 0.651 (95% confidence interval [CI], 0.592–0.711).

The apparent AUC for the score was 0.651 (95% CI, 0.592–0.711). Internal validation using 2,000 bootstrap resamples yielded an optimism estimate of 0.018 and an optimism‑corrected AUC of 0.633. Apparent calibration in the derivation cohort was acceptable, with a calibration intercept of 0.000, a calibration slope of 1.000, and a Brier score of 0.0313. The model-predicted revisit probabilities increased across score levels in a pattern consistent with observed revisit rates, as shown in Fig 2 and S2 Fig.

## Discussion

This study evaluated factors associated with unplanned revisits within 24 h among 2,774 ED visits resulting in discharge in which blood tests were performed and examined whether a simple five-item score could stratify revisit risk. The score demonstrated a graded association with revisit probability (Table 4, Fig 1), but its discrimination was modest, and sensitivity remained low at high specificity. These findings indicate that routinely collected index-visit information has limited ability to identify patients who will return very early after discharge, highlighting the inherent difficulty of predicting revisit behavior in a discharge-selected population.

Short-term revisits are influenced by a wide range of clinical and non-clinical factors [1]. Prior studies have shown that some return visits are associated with adverse events [14], whereas others reflect evolving symptoms, patient anxiety, or logistical issues rather than clinical deterioration [15–17]. This heterogeneity complicates prediction because the mechanisms underlying revisits are not uniform. In particular, revisits within 24 h after discharge may reflect a mixture of diagnostic uncertainty, symptom fluctuation, unmet expectations, or difficulty accessing outpatient care. Many of these factors are not captured by routine vital signs or laboratory data, which helps to explain the modest discrimination observed in this study.

Recent research further supports the inherent difficulty of revisit prediction. A scoping review of machine-learning models for ED revisit prediction reported substantial variability in performance and limited generalizability across institutions [19]. Even models using high-dimensional data or advanced algorithms often achieved only moderate discrimination. Another study developed the HANDLE-24 score to identify high-risk patients among those who revisited within 24 h after discharge, but its predictors included variables not universally available at the index visit [20]. These findings suggest that even sophisticated approaches face challenges when predicting revisits within 24 h after discharge, reinforcing the notion that routine index-visit data alone may be insufficient for reliable individual-level prediction.

In this context, the absence of clear differences in initial vital signs between the revisit and non-revisit groups is not surprising. Patients with markedly abnormal findings are typically admitted and therefore excluded from the discharge cohort. Previous studies have shown that abnormal discharge vital signs and physiologic deterioration during the ED stay are associated with adverse outcomes [3,8], but such signals may be attenuated when only discharged patients are analyzed. Clinician judgment has also been associated with short-term revisit risk [9], suggesting that unmeasured clinical impressions may capture aspects of vulnerability that are not reflected in routine measurements.

The laboratory variables included in the final score likely represent general physiologic or inflammatory burden rather than specific disease processes. C-reactive protein has been associated with early ED revisits [21], and hematologic indices such as lymphocyte percentage and red cell distribution width have been linked to outcomes in various acute care settings [22,23]. In our multivariable analysis, only no ambulance use and mean corpuscular volume ≤87 fL remained independently associated with revisit risk, suggesting that there was overlap among the selected items. The weighted model showed only minimal improvement over the equal-weight score, supporting the use of a simple additive structure for practical risk stratification. Nevertheless, the biological mechanisms linking hematologic indices such as MCV and RDW to very early ED revisits remain uncertain. Rather than representing direct causal factors, these variables may reflect underlying physiological vulnerability, residual clinical uncertainty at discharge, or characteristics specific to the present study population. Residual confounding cannot be excluded, and these associations should therefore be interpreted cautiously until confirmed in independent external cohorts.

The exploratory cutoff-screening procedure was data-driven, and although internal validation with 2,000 bootstrap resamples mitigated concerns about overfitting, the optimism-corrected AUC remained modest. This finding suggests that the predictive capacity of routinely collected variables is inherently constrained rather than limited by methodological choices. Although apparent calibration within the derivation cohort was acceptable (Fig 2, S2 Fig), good calibration does not compensate for limited discrimination when the goal is individual-level risk stratification.

The broader context of ED prediction research also provides insights. Scoring systems with high predictive performance have been reported in settings where the target condition and outcomes are pathophysiologically coherent. For example, a recent study developed a score for predicting poor outcomes in acute carbon monoxide poisoning and demonstrated high discrimination [24]. This illustrates that strong predictive performance is achievable when the clinical context and outcome are tightly linked, in contrast to the heterogeneous nature of ED revisits.

From a clinical perspective, the score developed in the present study may still have descriptive value. Although it should not be used as a stand-alone decision tool, it may help in characterizing gradients of revisit risk within a discharge-selected population and inform discussions about uncertainty at the time of discharge. However, its modest discrimination underscores that additional information, such as symptom trajectories, patient-reported concerns, social determinants, or follow-up accessibility, may be necessary to meaningfully improve prediction. Accordingly, the present score should not be used as a stand-alone tool for discharge decision-making. Rather, it should be viewed as a simple risk-stratification aid that may complement, but not replace, comprehensive clinical assessment. The low sensitivity observed at high-specificity thresholds further limits its utility for identifying individual high-risk patients. Importantly, this modest discrimination is itself informative, suggesting that routinely available discharge-time clinical and laboratory variables alone are insufficient to accurately predict very early ED revisits. Future prediction models with greater clinical utility will likely require additional patient-, disease-, and healthcare system-level information. The discrimination and calibration metrics used in the present study represent standard approaches for evaluating prediction models and should be interpreted in accordance with established methodological recommendations [25].

Finally, future research should explore approaches that integrate routinely collected clinical data with additional sources of information, such as natural-language processing of clinical notes, patient-reported symptoms, and contextual and social factors. Prospective evaluation will also be necessary to determine whether risk-stratification tools can meaningfully support clinical workflows or quality-improvement initiatives.

### Limitations

This study has several limitations. First, unplanned revisits were identified only within our institution, and revisits to other hospitals were not captured, which may have led to an underestimation of the true revisit incidence. However, such misclassification would not be expected to improve discrimination and likely reflects the inherent difficulty of predicting revisits within 24 h after discharge, using routine index‑visit data. Moreover, the performance of several predictors, particularly ambulance use, may depend on local emergency medical service utilization, physician practice patterns, and institutional discharge policies. Therefore, the present score should be interpreted within the context of the Japanese healthcare system until validated in other healthcare settings.

Second, the study population was restricted to discharged patients who underwent blood testing. This selection criterion may introduce spectrum bias, given that patients without laboratory testing were excluded and the findings may not generalize to all discharged ED patients. The included population represents encounters in which clinicians had sufficient diagnostic uncertainty to order laboratory tests, and the score should be interpreted within this context. Accordingly, the present score should not be generalized to all discharged emergency department patients. Because the study population reflects physician decision-making and institutional testing practices at a single center, its performance may differ in healthcare systems with different laboratory utilization patterns or discharge practices.

Third, vital signs were incompletely recorded, and laboratory testing was clinician‑directed rather than protocol‑based. Analyses were performed using available cases without imputation, and the missingness patterns are summarized in. S1 Table. Although this approach reflects real‑world practice, it may limit the stability of estimates for variables with lower availability. However, additional sensitivity analyses using complete-case analysis and multiple imputation produced very similar discrimination, suggesting that the study findings were robust to the method used for handling missing data.

Fourth, the exploratory cutoff‑screening procedure was data‑driven and may introduce instability. To mitigate this concern, the entire item‑selection and cutoff‑search process was repeated within each bootstrap sample during internal validation. Even after this correction, discrimination remained modest, suggesting that the predictive capacity of routinely collected variables is inherently limited rather than the result of overfitting alone. Accordingly, the selected cutoff values should be regarded as exploratory score-construction thresholds, and their stability and generalizability should be confirmed through external validation in independent cohorts.

Fifth, repeated visits by the same patient were treated as independent encounters in the primary analysis. A clustered sensitivity analysis accounting for within‑patient correlation produced similar results, but residual dependence cannot be entirely excluded.

Sixth, although the score demonstrated a graded association with revisit risk (Table 4, Fig 1), sensitivity remained low at high specificity. This limitation reflects the multifactorial nature of revisit behavior and underscores that routine index‑visit data alone are not sufficient for reliable individual‑level prediction.

Finally, this was a single‑center study conducted in a mixed urban–rural region of Japan. Patterns of ED utilization, revisit behavior, and laboratory testing differ across institutions and healthcare systems. External validation in diverse settings and prospective evaluation will be essential to determine the generalizability and practical utility of the score.

## Conclusion

In this study of 2,774 emergency department discharge visits with blood testing, a simple five‑item score demonstrated a graded association with unplanned revisits within 24 h, but its discrimination was modest and sensitivity remained low at high specificity. These findings indicate that routinely collected index‑visit information has limited ability to identify patients who will return very early after discharge, reflecting the multifactorial nature of revisit behavior. Although the score may help characterize gradients of revisit risk, it should not be used as a stand‑alone tool for clinical decision‑making. Future research should include external validation across diverse institutions and healthcare systems before broader clinical application and explore additional factors, such as longitudinal clinical trajectories, patient‑reported concerns, and contextual or social determinants, that might improve the identification of patients at risk for ED revisits within 24 h after discharge.

## Supporting information

S1 Fig Participant flow of emergency department discharge visits included in the derivation cohort. Among the 3,737 emergency department visits resulting in discharge assessed for eligibility in 2024, a total of 963 visits were excluded because no blood tests were performed at the index visit. The derivation cohort therefore comprised 2,774 visits, of which 91 had an unplanned revisit within 24 h after discharge and 2,683 did not.
(TIF)

S2 Fig Calibration plot of predicted versus observed 24-h revisit probability. Predicted probabilities were estimated using a logistic regression model with the total score entered as a continuous predictor. Points indicate the observed revisit rate for each score level, and error bars represent 95% confidence intervals. The diagonal dashed line indicates perfect calibration.
(TIF)

S1 Table Availability of vital signs and laboratory variables included in the between-group comparisons. (DOCX)

S2 Table Reference multivariable logistic regression model including all five score components. (DOCX)

S1 Dataset Anonymized dataset used for the analyses. The dataset contains de-identified individual visit-level data used for all analyses reported in this study.
(XLSX)

S1 TRIPOD Checklist TRIPOD checklist. (DOCX)
