# Supplementary material for: Development and internal validation of a simple risk stratification score for unplanned emergency department revisits within 24 hours after discharge: A retrospective observational study
Source: PLoS One. 2026 Sep 2;21(9):e0357575. doi: 10.1371/journal.pone.0357575 (PMC13537553; doi:10.1371/journal.pone.0357575)
Supplement: Supplementary file 1 [file pone.0357575.s003.docx]

**S1 Table. Availability of vital signs and laboratory variables included in the between-group comparisons.**

| **Variable** | **Available n (%)** |
| --- | --- |
| Blood pressure, mmHg |  |
| Systolic | 1,576 (56.8) |
| Diastolic | 1,567 (56.5) |
| Heart rate, /min | 1,291 (46.5) |
| Respiratory rate, /min | 1,423 (51.3) |
| SpO_2_, % | 1,573 (56.7) |
| Body temperature, °C | 1,602 (57.8) |
| Lymphocyte percentage, % | 2,678 (96.5) |
| Neutrophil fraction, % | 2,660 (95.9) |
| RBC, ×10^6^/µL | 2,770 (99.9) |
| RDW, % | 2,769 (99.8) |
| MCV, fL | 2,770 (99.9) |
| Hb, g/dL | 2,770 (99.9) |
| Cre, mg/dL | 2,770 (99.9) |
| BUN, mg/dL | 2,768 (99.8) |
| Na, mmol/L | 2,747 (99.0) |
| K, mmol/L | 2,747 (99.0) |
| Cl, mmol/L | 2,654 (95.7) |
| Alb, g/dL | 1,305 (47.0) |
| Lac, mmol/L | 403 (14.5) |
| CRP, mg/dL | 2,481 (89.4) |

Age, sex, ambulance transport, and time category were complete in all 2,774 visits and are therefore not shown.

Abbreviations: Alb, albumin; BUN, blood urea nitrogen; Cre, creatinine; CRP, C-reactive protein; Hb, hemoglobin; Lac, lactate; MCV, mean corpuscular volume; RBC, red blood cell count; RDW, red cell distribution width (RDW-CV); SpO_2_, oxygen saturation
