# Supplementary material for: Development and internal validation of a simple risk stratification score for unplanned emergency department revisits within 24 hours after discharge: A retrospective observational study
Source: PLoS One. 2026 Sep 2;21(9):e0357575. doi: 10.1371/journal.pone.0357575 (PMC13537553; doi:10.1371/journal.pone.0357575)
Supplement: Supplementary file 2 [file pone.0357575.s004.docx]

**S2 Table. Reference multivariable logistic regression model including all five score components.**

| **Parameter** | **Adjusted OR** | **95% CI** | ***P*-value** |
| --- | --- | --- | --- |
| No ambulance transport | 2.05 | 1.33–3.15 | 0.001 |
| CRP, mg/dL | 1.55 | 0.98–2.44 | 0.058 |
| Lymphocyte percentage, % | 1.31 | 0.83–2.07 | 0.238 |
| RDW, % | 1.33 | 0.86–2.05 | 0.199 |
| MCV, fL | 1.65 | 1.03–2.63 | 0.036 |
