# Supplementary material for: Development and internal validation of a simple risk stratification score for unplanned emergency department revisits within 24 hours after discharge: A retrospective observational study
Source: PLoS One. 2026 Sep 2;21(9):e0357575. doi: 10.1371/journal.pone.0357575 (PMC13537553; doi:10.1371/journal.pone.0357575)
Supplement: Supplementary file 3 [file pone.0357575.s006.docx]

**TRIPOD checklist**

**1. Title**
Checklist item: Identify the study as developing a multivariable prediction model, the target population, and the outcome.
Reported in manuscript: Title page; the title identifies the study as the development and internal validation of a multivariable prediction model for unplanned emergency department revisits within 24 after discharge and states that the study is retrospective and observational.

**2. Abstract**
Checklist item: Provide a summary of objectives, study design, setting, participants, sample size, predictors, outcome, statistical analysis, results, and conclusions.
Reported in manuscript: Abstract; the abstract summarizes the background, single-center retrospective design, 2,774 emergency department visits resulting in discharge, 24-h unplanned revisit outcome, exploratory predictor screening, bootstrap internal validation, main results, and conclusion.

**3a. Background and rationale**
Checklist item: Explain the medical context and rationale for developing the prediction model, including whether it is diagnostic or prognostic and references to existing models.
Reported in manuscript: Introduction; the manuscript explains the clinical importance and limitations of short-term revisit prediction, discusses heterogeneous outcome meaning, and cites prior conventional and machine-learning studies.

**3b. Objectives**
Checklist item: Specify the objectives, including that the study describes the development of a prediction model.
Reported in manuscript: Objective; the manuscript states that the aim was to develop and internally validate a simple score for unplanned emergency department revisits within 24 h after discharge.

**4a. Source of data**
Checklist item: Describe the study design or source of data used for model development.
Reported in manuscript: Ethical considerations; Study design and participants; the manuscript describes a single-center retrospective observational study using existing clinical information from the institutional emergency department.

**4b. Key study dates**
Checklist item: Specify key study dates, including start and end of accrual and, if applicable, end of follow-up.
Reported in manuscript: Study design and participants; the study period is 2024, including all emergency department visits in that year, and the outcome window is within 24 h after discharge.

**5a. Study setting**
Checklist item: Specify key elements of the study setting, such as emergency care, number of centres, and location.
Reported in manuscript: Study design and participants; the setting is a single emergency department at one institution in Kochi, Japan.

**5b. Eligibility criteria**
Checklist item: Describe eligibility criteria for participants.
Reported in manuscript: Study design and participants; the manuscript includes emergency department discharge visits with blood tests at the index visit and excludes visits without blood testing and visits resulting in admission, transfer, or transport to another facility. The intended scope of the model is therefore emergency department visits resulting in discharge in which blood testing had been performed at the index visit, rather than all discharged patients.

**5c. Treatments received**
Checklist item: Give details of treatments received, if relevant.
Reported in manuscript: Not applicable; this is a model development study and treatment exposure is not a central model component.

**6a. Outcome**
Checklist item: Clearly define the outcome that is predicted, including how and when it was assessed.
Reported in manuscript: Outcome definition; the outcome is an unplanned revisit to the same emergency department within 24 h after discharge, identified from institutional visit records and adjudicated by two physicians, using administrative records and chart review.

**6b. Blinding of outcome assessment**

Checklist item: Report any actions to blind assessment of the outcome to be predicted.

Reported in manuscript: Outcome definition; the manuscript states that no formal blinding procedure was used for adjudication of outcomes.

**7a. Predictors**
Checklist item: Clearly define all predictors used in developing the prediction model, including how and when they were measured.
Reported in manuscript: Data collection and candidate predictors; Handling of vital signs and laboratory variables; the manuscript defines mode of arrival, initial vital signs, and laboratory variables obtained at the index emergency department visit.

**7b. Blinding of predictor assessment**

Checklist item: Report any actions to blind assessment of predictors for the outcome and other predictors.

Reported in manuscript: Data collection and candidate predictors; the manuscript states that predictor values were extracted from routine index-visit records and that no formal blinding procedure was used for predictor assessment.

**8. Sample size**

Checklist item: Explain how the study size was arrived at.

Reported in manuscript: Study design and participants; the manuscript states that no formal sample size calculation was performed and that all consecutive eligible emergency department discharge visits during the study period were included.

**9. Missing data**
Checklist item: Describe how missing data were handled.
Reported in manuscript: Handling of vital signs and laboratory variables; Availability of recorded variables; Supplementary Table S1; the manuscript states that no imputation was performed for predictor-level analyses, that analyses used the recorded values that were available in routine care, and that unmeasured final score components were coded as “absent” for calculation of the final additive score

**10a. Statistical analysis methods**
Checklist item: Describe how predictors were handled in the analyses.
Reported in manuscript: Statistical analysis; continuous candidate predictors were screened across prespecified percentile points within the observed distribution, specifically from the 5th to the 95th percentiles in 5-percentile increments, dichotomized at the cutoff yielding the largest odds ratio, and then considered for additive score construction. Candidate items were screened exploratorily from the routinely available index-visit variables, and final item retention was based on the exploratory cutoff-screening procedure rather than on descriptive *P*-values alone.

**10b. Statistical analysis methods**
Checklist item: Specify the type of model, all model-building procedures including any predictor selection, and method for internal validation.
Reported in manuscript: Statistical analysis; the manuscript describes logistic regression, exploratory cutoff screening, additive score derivation, weighted-model comparison, and bootstrap internal validation with 2,000 resamples.

**10c. Statistical analysis methods**
Checklist item: For validation, describe how the predictions were calculated.
Reported in manuscript: Not applicable; this is an exploratory study without an external validation dataset.

**10d. Statistical analysis methods**
Checklist item: Specify all measures used to assess model performance and, if relevant, to compare multiple models.
Reported in manuscript: Statistical analysis; Discrimination, calibration, and internal validation; Comparison with a weighted model; the manuscript reports area under the curve, optimism-corrected area under the curve, sensitivity, specificity, positive predictive value, negative predictive value, calibration intercept, calibration slope, and Brier score.

**11. Risk groups**
Checklist item: Provide details on how risk groups were created, if done.
Reported in manuscript: Threshold performance; Table 4; the manuscript reports score categories 0–5 and selected cutoffs of ≥2 and ≥4.

**12. Development vs validation**
Checklist item: For validation, identify any differences from the development data in setting, eligibility criteria, outcome, and predictors.
Reported in manuscript: Not applicable; the study aim is model development with internal validation only.

**13a. Participants**
Checklist item: Describe the flow of participants, including the number of participants with and without the outcome and, if applicable, a summary of the follow-up time.
Reported in manuscript: Study cohort; Supplementary Figure S1; the manuscript reports 3,737 assessed discharge visits, 963 excluded without blood testing, 2,774 included visits, 91 unplanned revisits, and 2,683 non-events.

**13b. Participants**
Checklist item: Describe the characteristics of the participants, including the number of participants with missing data for predictors and outcome.
Reported in manuscript: Table 1; Table 2; Supplementary Table S1; the manuscript reports baseline characteristics, between-group comparisons, and the availability of vital signs and laboratory variables.

**14a. Model development**
Checklist item: Specify the number of participants and outcome events in each analysis.
Reported in manuscript: Statistical analysis; Results; the manuscript gives 2,774 visits, 91 unplanned revisits, available-case sample sizes for the final score components, and states that the final score could be calculated for all 2,774 included visits because unmeasured final score components were coded as “absent” rather than imputed.

**14b. Model development**
Checklist item: If done, report the unadjusted association between each candidate predictor and outcome.
Reported in manuscript: Between-group comparisons; Table 2; Table 3; the manuscript reports unadjusted comparisons and univariable associations for retained predictors.

**15a. Model specification**
Checklist item: Present the full prediction model to allow predictions for individuals.
Reported in manuscript: Table 3; Table 4; the manuscript presents the retained score components, dichotomization thresholds, and score-level event rates.

**15b. Model specification**

Checklist item: Explain how to use the prediction model.

Reported in manuscript: Candidate predictors and score derivation; Table 3; Table 4; the manuscript explains that 1 point was assigned for each retained dichotomized item present, yielding a total score ranging from 0 to 5, with higher scores indicating higher revisit risk.

**16. Model performance**
Checklist item: Report performance measures with confidence intervals.
Reported in manuscript: Results; Figure 2; Supplementary Figure S2; Table 4; the manuscript reports apparent and optimism-corrected discrimination, calibration measures, and threshold performance with confidence intervals where applicable.

**17. Model-updating**
Checklist item: If done, report the results from any model updating.
Reported in manuscript: Not applicable; no model updating was performed.

**18. Limitations**
Checklist item: Discuss any limitations of the study.
Reported in manuscript: Limitations; the manuscript discusses single-center design, outcome heterogeneity, the inability to capture revisits to other hospitals, documentation-dependent adjudication, selection based on blood testing, visit-level analysis, exploratory cutoffs, potential bias related to missing predictor data, and healthcare-system dependence affecting model generalizability.

**19a. Interpretation**
Checklist item: For validation, discuss the results with reference to performance in the development data and any other validation data.
Reported in manuscript: Not applicable; this is not an external validation study.

**19b. Interpretation**
Checklist item: Give an overall interpretation of the results, considering objectives, limitations, results from similar studies, and other relevant evidence.
Reported in manuscript: Discussion; the manuscript interprets the score as showing a graded association but limited predictive performance and places the findings in the context of prior research on emergency department revisits.

**20. Implications**
Checklist item: Discuss the potential clinical use of the model and implications for future research.
Reported in manuscript: Discussion; Conclusion; the manuscript emphasizes the limited predictive performance of routinely available discharge-time information, the role of the proposed score as a simple risk-stratification aid rather than a stand-alone clinical decision tool, the need for external validation across diverse institutions and healthcare systems, and the incorporation of additional clinical, patient-level, and contextual information in future prediction models.

**21. Supplementary information**
Checklist item: Provide information about the availability of supplementary resources, such as study protocol, web calculator, and data sets.
Reported in manuscript: Supplementary Figure S1; Supplementary Figure S2; Supplementary Tables S1 and S2; anonymized study dataset; TRIPOD checklist; Availability of data and materials; the manuscript provides supplementary figures and tables, a completed TRIPOD checklist, and an anonymized dataset as Supporting Information.

**22. Funding**
Checklist item: Give the source of funding and the role of the funders for the present study.
Reported in manuscript: Declarations; the manuscript states that the work was supported by The Kochi Medical School Hospital President’s Discretionary Grant.
